# CYP1B1 as a therapeutic target in cardio-oncology

**DOI:** 10.1042/CS20200310

**Published:** 2020-11-13

**Authors:** Alexa N. Carrera, Marianne K.O. Grant, Beshay N. Zordoky

**Affiliations:** 1 Department of Experimental and Clinical Pharmacology, College of Pharmacy, University of Minnesota, Minneapolis, MN, United States of America; 2 Augsburg University, Minnesota, Minneapolis, MN, United States of America

**Keywords:** Cardio-Oncology, chemotherapy, CYP1B1, Phytochemicals, radiation therapy

## Abstract

Cardiovascular complications have been frequently reported in cancer patients and survivors, mainly because of various cardiotoxic cancer treatments. Despite the known cardiovascular toxic effects of these treatments, they are still clinically used because of their effectiveness as anti-cancer agents. In this review, we discuss the growing body of evidence suggesting that inhibition of the cytochrome P450 1B1 enzyme (CYP1B1) can be a promising therapeutic strategy that has the potential to prevent cancer treatment-induced cardiovascular complications without reducing their anti-cancer effects. CYP1B1 is an extrahepatic enzyme that is expressed in cardiovascular tissues and overexpressed in different types of cancers. A growing body of evidence is demonstrating a detrimental role of CYP1B1 in both cardiovascular diseases and cancer, via perturbed metabolism of endogenous compounds, production of carcinogenic metabolites, DNA adduct formation, and generation of reactive oxygen species (ROS). Several chemotherapeutic agents have been shown to induce CYP1B1 in cardiovascular and cancer cells, possibly via activating the Aryl hydrocarbon Receptor (AhR), ROS generation, and inflammatory cytokines. Induction of CYP1B1 is detrimental in many ways. First, it can induce or exacerbate cancer treatment-induced cardiovascular complications. Second, it may lead to significant chemo/radio-resistance, undermining both the safety and effectiveness of cancer treatments. Therefore, numerous preclinical studies demonstrate that inhibition of CYP1B1 protects against chemotherapy-induced cardiotoxicity and prevents chemo- and radio-resistance. Most of these studies have utilized phytochemicals to inhibit CYP1B1. Since phytochemicals have multiple targets, future studies are needed to discern the specific contribution of CYP1B1 to the cardioprotective and chemo/radio-sensitizing effects of these phytochemicals.

## Introduction

Cancer survivorship has significantly increased over the past two decades, thanks to advanced diagnosis and treatment of different types of cancers. Currently, there are more than 15 million cancer survivors in the United States and this number is expected to increase due to the continued improvement of diagnostics, therapeutics, and care models [1]. Although the increased survivorship is a cause for celebration, two-thirds of cancer survivors experience at least one late adverse effect [2]. Cardiovascular disease is the second highest cause of mortality in cancer survivors, after secondary malignancy. The cardiovascular toxicity of cancer treatments has been increasingly recognized as a critical issue in the care of cancer survivors. Therefore, cardio-oncology has emerged as a clinical subspecialty with an ultimate goal to mitigate cardiovascular complications in cancer patients and survivors [3,4]. Cardiovascular complications have been reported in cancer patients and survivors who received different types of cancer treatments including anthracyclines, monoclonal antibodies, alkylating agents, tyrosine kinase inhibitors, immune checkpoint inhibitors, proteasome inhibitors, and radiation, as reviewed in [5]. Despite the known cardiovascular toxic effects of these treatments, they are still clinically used because of their effectiveness as anti-cancer agents. Protection against cancer treatment-induced cardiotoxicity is challenging, because shared mechanistic pathways may contribute to both the tumor suppressive and the cardiotoxic effects of cancer treatments. For instance, anthracycline-induced apoptotic cell death is a shared pathway for the anti-cancer and cardiotoxic effects of anthracyclines [6]. Cardioprotective agents that have non-selective anti-apoptotic effects will likely inhibit the anti-cancer effects of anthracyclines. Likewise, novel immune checkpoint inhibitors activate the immune system to fight the cancer; however, this may lead to immune-mediated myocarditis [7]. In this scenario, indiscriminate immunosuppression may protect the heart, but will likely undermine the anti-cancer effects of these agents. Therefore, there is a critical need to identify therapeutic targets that have the potential to prevent cancer treatment-induced cardiovascular complications without reducing their anti-cancer effects.

Cytochrome P450 1B1 (CYP1B1) is a monooxygenase enzyme involved in the metabolism of a variety of xenobiotics and endogenous compounds [8]. In this review, we will discuss the growing body of evidence suggesting that CYP1B1 can be a promising therapeutic target in cardio-oncology. First, we will give a brief overview of the expression, regulation, and metabolic activity of CYP1B1. Second, we will briefly discuss the role of CYP1B1 in both cardiovascular diseases and cancer. Then, we will summarize the existing literature showing how CYP1B1 is involved in the cardiovascular toxicity of different cancer treatments and the potential cardiovascular protective effects of CYP1B1 inhibitors. In parallel, we will also discuss the role of CYP1B1 inhibitors in preventing resistance to cancer treatments to highlight that CYP1B1 inhibition may not only prevent cardiovascular toxicity, but also augment the anti-cancer effects of different cancer treatments. Importantly, we will discuss how CYP1B1-mediated signaling pathways may have divergent effects of the cardiovascular tissues and the cancer. At last, we will comment on the challenges that face clinically targeting CYP1B1 and highlight future research directions.

## CYP1B1

CYP1B1 is a member of the CYP1 gene family which also includes CYP1A1 and CYP1A2. A novel cytochrome P450 enzyme (P450-EF) was first purified from 2,3,7,8-tetrachlorodibenzo-*p*-dioxin (TCDD)-treated mouse embryonic fibroblasts [9]. In 1994, P450-EF was identified and cloned as the mouse *Cyp1b1* [10]. In parallel, human *CYP1B1* was first cloned from TCDD-treated human epidermal keratinocytes [11]. *CYP1B1* showed approximately 40% homology with both *CYP1A1* and *CYP1A2* [12]. The human *CYP1B1* gene is located on chromosome 2 and contains three exons and two introns [13]. Mouse and rat orthologs of *CYP1B1* have also been cloned and characterized [12]. Although each of these orthologs has an mRNA of 5.2 kb and a predicted protein of 543 amino acids [12], they show significant species differences in their regulation, metabolic activity, and tissue-specific distribution [10,12–14].

### Expression

Unlike most cytochrome P450 enzymes, CYP1B1 expression has not been detected in the human liver; however, it is expressed primarily in extrahepatic tissues [8]. Of importance in cardio-oncology, CYP1B1 has been shown to be expressed in cardiovascular tissues and overexpressed in malignant tumors. Indeed, CYP1B1 has been detected at the mRNA and protein levels in cardiovascular tissues of human and experimental animals [15]. CYP1B1 mRNA and protein have been detected in the rat and mouse heart and in the cardiac-derived H9c2 cells [16–19]. In addition to the myocardial tissues, CYP1B1 has been detected in the vasculature in both vascular smooth muscle cells and endothelial cells [20–25]. Intriguingly, CYP1B1 has been shown to be overexpressed in malignant tumor tissues [26], particularly in hormone-responsive tissues such as prostate [27], breast [28], and ovarian cancers [29,30]. Additional immunohistochemical studies showed that CYP1B1 protein expressions were detected in 53 out of 62 samples of the extrahepatic tissue. Among these 62 samples include human brain cortex tissues, kidney tissues, and lymphoid, prostate, cervix, uterus, oocytes, bone marrow, epithelial, smooth muscle cells, and ovary cells [22,31–33].

### Regulation

The *CYP1B1* gene is transcriptionally induced by polycyclic aromatic hydrocarbons (e.g. TCDD) via the Aryl hydrocarbon Receptor (AhR) complex, which is a transcriptional factor that regulates CYP1A1 and CYP1B1 [11,12]. Xenobiotic-responsive elements (XREs) have been identified in the 5′ regulatory region of the *CYP1B1* gene [34]. Induction of the human, rat and mouse *CYP1B1* gene expression by AhR agonists has been well-documented in a variety of cell types [35–39]. In addition, the AhR is highly expressed in the heart [40], and activation of the AhR has been shown to induce CYP1B1 in cardiovascular tissues. For instance, concentrated ambient particles induce CYP1B1 mRNA in rat hearts [41]. Similarly, benzo(a)pyrene, a component of cigarette smoke, has been shown to induce CYP1B1 in the rat heart [42]. Conversely, AhR antagonists inhibit constitutive CYP1B1 expression [43]. Interestingly, CYP1B1 has been shown to be constitutively expressed in the hearts of both control and AhR-deficient mice, which implies the involvement of other pathways that regulate cardiac CYP1B1 [44].

AhR-independent up-regulation of CYP1B1 may be mediated by inflammation, estrogen signaling or other endogenous compounds. Inflammation has been shown to down-regulate most cytochrome P450 enzymes of the CYP1, CYP2, and CYP3 families [45,46]. In contrast, a few isoforms are up-regulated by inflammation such as CYP4F enzymes and CYP1B1 [46,47]. Specifically, the inflammatory cytokine interleukin-6 (IL-6) has been shown to induce CYP1B1 via miR27b in colorectal and breast cancer cells [48,49]. Tumor necrosis factor-α (TNF-α) has also been shown to up-regulate CYP1B1 via a p38-mediated mechanism in rat liver epithelial cells [32,50]. CYP1B1 is also up-regulated by 17β-estradiol through Estrogen Receptor α (ERα) [51]. G protein estrogen receptor (GPER) is also involved in CYP1B1 regulation [52]. Leptin and prostaglandin E2 have also been shown to up-regulate CYP1B1 expression through ligand-independent activation of the ERα pathway in MCF-7 breast cancer cells [53,54]. Other pathways that may play a role in CYP1B1 regulation include: the peroxisome proliferator-activated α (PPARα) in MCF-7 and HCT116 cells [55,56], the Wnt/β-catenin signaling pathway in endothelial cells and adreno-corticotropic hormone (ACTH) via cAMP in adrenal cells [36,57–59].

### Metabolic activity

**Figure 1:**
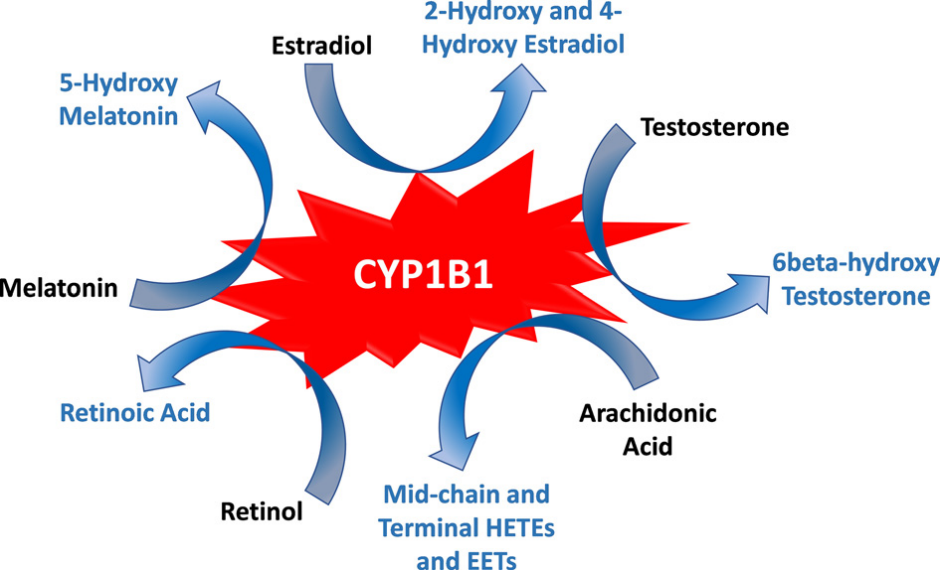
CYP1B1 is a central player in the metabolism of endogenous compounds CYP1B1 metabolizes estradiol, testosterone, arachidonic acid, retinol, and melatonin to the biologically active metabolites: 2- and 4-hydroxyestradiol, 6β-hydroxytestosterone, mid-chain and terminal hydroxyeicosatetraenoic acids (HETEs), epoxyeicosatrienoic acids (EETs), retinoic acid, and 5-hydroxymelatonin, respectively.

CYP1B1 has been shown to metabolize both endogenous (Figure 1) and exogenous compounds. CYP1B1 plays an important role in steroid metabolism, as reviewed in [60]. Estradiol is the preferred substrate for CYP1B1, followed by progesterone, then testosterone [61]. CYP1B1 metabolizes estradiol and estrone to their respective 4-hydroxy and 2-hydroxy metabolites [62–64]. Although at a lower activity, CYP1B1 has also been found to metabolize estradiol to 15α-, 6α-, 16α-, and 6β-hydroxy metabolites [61]. The 4-hydroxy-estradiol can be transformed to semiquinones and quinones that can form DNA adducts resulting in oncogenic effects [65,66] and undergo redox cycling to generate reactive oxygen species (ROS) [67]. Intriguingly, 4-hydroxyestradiol has been shown to up-regulate CYP1B1 in human mammary epithelial MCF-10A cells in a positive feedback loop [68]. Regarding androgen metabolism, CYP1B1 catalyzes the 6β-hydroxylation and 16α-hydroxylation of testosterone [61,63].

CYP1B1 is also involved in arachidonic acid metabolism. Arachidonic acid is metabolized by cytochrome P450 monooxygenases to different regioisomers of epoxyeicosatrienoic acids (EETs) and hydroxyeicosatetraenoic acids (HETEs) [15,69,70]. Human and rat CYP1B1 orthologs have been reported to metabolize arachidonic acid primarily to mid-chain HETEs, while the main metabolites of the mouse ortholog were EETs [71,72]. CYP1B1-mediated production of mid-chain HETEs have been implicated in the pathogenesis of cardiac hypertrophy [72,73], and in doxorubicin (DOX)-induced cardiotoxicity [74]. In addition to steroid and arachidonic acid metabolism, both mouse and human CYP1B1 orthologs have been shown to oxidize retinol to retinal and retinal to retinoic acid [71,75]. Melatonin can also be metabolized to 6-hydroxymelatonin or converted back into N-acetylserotonin by CYP1B1 [76,77]. Regarding xenobiotic metabolism, CYP1B1 binds planar polyaromatic ring systems such as polyaromatic hydrocarbons to catalyze a monooxygenation step to produce carcinogenic metabolites [78,79]. CYP1B1 has also been shown to metabolize clinically relevant drugs such as theophylline, caffeine, and flutamide [80,81].

### Role of CYP1B1 in cardiovascular diseases

We and others have demonstrated a significant role of CYP1B1 in the pathogenesis of cardiovascular diseases, most remarkably in cardiac hypertrophy and hypertension (Table 1). El-Kadi and colleagues demonstrated that cardiac CYP1B1 expression was up-regulated in different models of cardiac hypertrophy induced by isoproterenol [82–84], pressure overload [85,86], angiotensin II [73], and polycyclic aromatic hydrocarbons [42,87]. Additionally, heavy metal-induced cardiotoxicity has been associated with up-regulation of cardiac CYP1B1 [88–90]. The induction of CYP1B1 in these studies was associated with a perturbation in cardiac arachidonic acid metabolism with generation of more terminal and mid-chain HETEs. Importantly, inhibition of CYP1B1-mediated mid-chain HETEs production has been shown to prevent cardiac hypertrophy in male rats [73,86]. Confirming the causative role of CYP1B1 in developing cardiac hypertrophy, overexpression of CYP1B1 using CRISPR technology has been shown to induce cellular hypertrophy in the cardiac-derived RL-14 cells [72]. Inhibition of CYP1B1 has also been recently shown to prevent uremic toxins-induced cardiac hypertrophy [91]. Additionally, 2-methoxyestradiol, a specific CYP1B1 inhibitor, protected against pressure overload-induced cardiac hypertrophy via antioxidant and anti-inflammatory properties [86]. Similarly, Malik and colleagues have demonstrated an important role of CYP1B1 in hypertension and hypertension-associated pathophysiology [92]. Intriguingly, they have shown a sexually dimorphic role of CYP1B1 where CYP1B1 played a detrimental role in male rodents [93–95], while it had a protective effect in females [96,97]. The detrimental effects of CYP1B1 in male rodents have been attributed to CYP1B1-mediated production of 6β-hydroxytestosterone which was shown to exacerbate angiotensin II-induced hypertension [93], renal dysfunction [94], and vascular changes [95]. On the other hand, the protective effects of CYP1B1 in female rodents have been attributed to CYP1B1-mediated metabolism of estrogen to 2-methoxyestradiol [96,97]. Furthermore, CYP1B1 has been shown to contribute to the development of atherosclerosis, hypertension, and angiotensin II-induced aortic aneurysm in male apolipoprotein E-deficient mice [98,99]. *In vitro* studies have suggested the contribution of CYP1B1-mediated formation of genotoxic metabolites and DNA adducts in the development of atherosclerosis by polyaromatic hydrocarbons [100,101].

**Table 1:** Role of CYP1B1 in cardiovascular diseases

Cardiovascular pathology	Model	Effect on CYP1B1 expression	Effect of CYP1B1 inhibition	References
Cardiac hypertrophy	Isoproterenol-induced cardiac hypertrophy in male SD rats	Up-regulation of CYP1B1 gene and protein expression in the heart	Not reported	[72,83]
	Isoproterenol-induced cellular hypertrophy in RL-14 cells	Induction of *CYP1B1* gene expression	Inhibition of CYP1B1 by TMS or siRNA ameliorated isoproterenol-induced cellular hypertrophy	[72]
	Abdominal aortic constriction in male SD rats	Increase in the protein expression of CYP1B1	2-ME inhibited left ventricular hypertrophy via antioxidant and anti-inflammatory mechanisms	[85,86]
	Angiotensin II-induced cellular hypertrophy in RL-14 and H9c2 cells	Induction of the protein expression of CYP1B1 and increased formation of its associated mid-chain HETEs	Inhibition of CYP1B1 by TMS, resveratrol, fluconazole or 19-HETE attenuated angiotensin II-induced cellular hypertrophy	[73,116–118]
	Angiotensin II-induced cardiac hypertrophy in male SD rats	Induction of CYP1B1 protein expression, but no effect on *CYP1B1* gene expression	Inhibition of CYP1B1 by TMS or 19-HETE ameliorated angiotensin II-induced cardiac hypertrophy	[73,118]
Hypertension	DOCA salt-induced hypertension in male Sprague–Dawley rats	No significant effect on CYP1B1 expression or activity	Inhibition of CYP1B1 by TMS reduced blood pressure, ameliorated cardiovascular and renal hypertrophy, and prevented vascular reactivity and endothelial dysfunction	[119]
	Male SHR rats	Higher CYP1B1 activity in the aorta, heart and kidney of SHRs as compared with control WKY rats	Inhibition of CYP1B1 by TMS reduced blood pressure, decreased vascular reactivity, cardiovascular hypertrophy, endothelial and renal dysfunction, and cardiac and renal fibrosis	[120]
	Angiotensin II-induced hypertension in intact male and OVX female mice	Not reported	Inhibition of CYP1b1 with 2-ME reduced blood pressure in ovariectomized female and intact male mice	[121]
	Angiotensin II-induced hypertension in male mice	Increased renal Cyp1b1 activity, increased 12-HETE and 20-HETE metabolites	*Cyp1b1* gene disruption reduced blood pressure and renal damage	[122]
	Angiotensin II-induced hypertension in female mice	Increased cardiac Cyp1b1 protein expression and catalytic activity	*Cyp1b1* gene disruption exacerbated hypertension and renal damage	[97,123]
	Angiotensin II-induced hypertension in male mice	Increased cardiac cytochrome P450 1B1 activity and plasma levels of 6β-hydroxytestosterone	*Cyp1b1* gene disruption mitigated angiotensin II-induced increase in systolic blood pressure and associated cardiac hypertrophy and fibrosis	[93]
Atherosclerosis	ApoE-deficient male mice on atherogenic diet	Increased cardiac Cyp1b1 activity	Cyp1b1 inhibition by TMS or gene disruption ameliorated atherosclerosis, and reduced blood pressure, endothelial dysfunction, oxidative stress and plasma lipids	[99]
Aortic aneurysm	Angiotensin II-induced aortic aneurysm in male ApoE-deficient mice	Not reported	Cyp1b1 inhibition by TMS or *Cyp1b1* gene disruption minimized aortic aneurysms via reduction in oxidative stress and inflammation	[98]
Heavy metal-induced cardiotoxicity	Acute arsenic toxicity in male C57Bl/6 mice	Induction of CYP1B1 gene expression	Not reported	[90]
	Acute mercury toxicity in male C57Bl/6 mice	Induction of cardiac *CYP1b1* gene expression	Not reported	[89]
	Cadmium-induced toxicity in newborn chicks	Increase in total CYP1B1 expression	Not reported	[88]

Abbreviations: ApoE, apolipoprotein E; DOCA, deoxycorticosterone acetate; SHR, spontaneously hypertensive rat; TMS, 2,4,3′,5′-tetramethoxystilbene; WKY, Wistar–Kyoto rat; 2-ME, 2-methoxyestradiol.

Several studies have reported the expression of other cytochrome P450 enzymes in cardiovascular tissues including human heart, aorta, and coronary arteries [16,102,103], as previously reviewed [15,104]. CYP2J2 is the most highly expressed cytochrome P450 enzyme in human cardiovascular tissues [103]. CYP2J2 metabolizes arachidonic acid to EETs which exhibit cardioprotective and anti-inflammatory properties [105,106]. Although overexpression of CYP2J2 has been shown to protect against anthracycline-induced cardiotoxicity in transgenic mice [107], CYP2J2-mediated EETs may promote tumor progression and metastasis [108,109]. Therefore, CYP2J2 may not be a reasonable therapeutic target in cardio-oncology. On the other hand, CYP1A1 has been shown to contribute to both anthracycline-induced cardiotoxicity [110–113] and tumor progression and survival of cancer cells [114,115]. Therefore, similar to CYP1B1, CYP1A1 may also be a reasonable therapeutic target in cardio-oncology. Taken together, isoform-specific targeting of cytochrome P450 enzymes is critical in cardio-oncology, since different isoforms may have opposing effects on the cancer or the cardiovascular system.

### Role of CYP1B1 in cancer

The human CYP1B1 enzyme is overexpressed in numerous tumors compared with normal tissues [124]. For instance, immunohistochemistry reports showed high CYP1B1 mRNA and protein levels in prostate tumors, mammary tumors and peritumor benign tissues, and ovarian cancer tissues [30]. Similarly, CYP1B1 was shown to be expressed in eight different cell lines that represent four tumor tissues, with the highest expression levels manifested in HeLa, SKOV-3, and MDA-MB-231 cells, respectively [124]. CYP1B1 overexpression has been associated with the increase in cancer risk via pro-inflammatory cytokines, metastasis, and disturbance in the regulation of cell proliferation, migration, and differentiation [125–128]. Additionally, CYP1B1 overexpression is also associated with increased tumor size, a higher tumor grade, frequent lymph node metastasis, and lymphovascular invasion [125]. In cancer cells, CYP1B1 is thought to play a role in the bioactivation of xenobiotics, metabolism of steroid hormones, and the production of multiple pro-inflammatory and pro-angiogenic factors [26]. The detrimental effects of CYP1B1 have been demonstrated not only in cancer cells, but also in other cell types, including fibroblasts, endothelial cells, pericytes, and immune cells which constitute the tumor micro-environment, as reviewed in [26]. This is especially important, considering the crucial role of the tumor micro-environment in cancer progression and metastasis [26]. For instance, in endothelial cells, CYP1B1 was observed to promote endothelial nitric oxide synthase (eNOS) expression as well as nitric oxide levels, responsible for the many inflammatory and angiogenesis effects important for cancer progression [129,130].

The exact mechanisms of CYP1B1 overexpression in cancer cells and tumors are not fully elucidated. However, CYP1B1 is particularly overexpressed in hormone-related or estrogen-dependent cancers, such as breast, ovarian, and prostate cancers [126,131]. This can be attributed to CYP1B1 involvement in the metabolism of estrogen, progesterone, testosterone, and other steroid-related hormones. CYP1B1-mediated metabolism of these hormones can result in the generation of genotoxic metabolites and oxidative damage [30,132]. Additionally, pro-inflammatory cytokines such as TNF-α and IL-6 have been especially known to induce the expression of CYP1B1 [49,133]. The mRNA and protein levels of the AhR and CYP1B1 are higher in inflammatory breast cancer tissues [126]. CYP1B1 role in carcinogenesis may be attributed to its ability to metabolize polycyclic aromatic hydrocarbons and activate pro-carcinogens into DNA-reactive metabolites [134]. Additionally, CYP1B1 converts melatonin into N-acetylserotonin which then activates tyrosine receptor kinase B (TrkB), eventually leading to breast cancer cell survival and migration [77]. WY-14643, a PPARα agonist, has been shown to increase the protein and mRNA levels of CYP1B1 in MCF-7 cells via PPARα-dependent mechanism, playing a critical role in the progression of human breast cancer [55]. Another way in which CYP1B1 has been shown to play a role in cancer development is by enhancing the invasion of MCF-7 and MCF-10A cells. CYP1B1 has been shown to induce epithelial–mesenchymal transition (EMT) and up-regulates several transcription factors involved in cell growth and metastasis via Sp1 induction [126]. A major metabolite generated by CYP1B1, 4-hydroxyestradiol, also mediates many oncogenic events in cells via the formation of DNA adducts [126,128]. Intriguingly, overexpression of CYP1B1 in tumors can also be attributed to its induction by chemotherapeutic agents and radiation therapy, as summarized in Table 2 and discussed in more detail in subsequent sections of the review.

**Table 2:** Effect of cancer treatments on CYP1B1 expression

Agent	Model	Dose/concentration	Effect on CYP1B1	References
Cyclophosphamide	HL-60S and HL-60R human promyelocytic leukemia sensitive (S) and resistant (R) cell lines	100 and 500 µg/ml 1, 2, 3 days	Concentration-dependent inhibition of gene expression	[175]
Doxorubicin (DOX)	Zebrafish	100 μM 40 h	Induction of gene and protein expression	[113]
	C57Bl/6 male and female mice	20 mg/kg single dose 1 day, 6 day	Induction of gene expression in the heart of male mice only	[19]
	Sprague–Dawley male rats	3 mg/kg × 5 doses (over 2-week-period) 1 day post	Induction of gene expression in the heart Increased mid-chain HETEs	[74]
	Sprague–Dawley male rats	2.5 mg/kg × 6 doses (over 2-week-period) 14 days post	Induction of gene expression in the liver and kidney	[168]
	Sprague–Dawley male rats	15 mg/kg single dose 1 day post	Induction of gene expression in the liver and kidney	[167]
	Sprague–Dawley male rats	15 mg/kg single dose 1 day post	Induction of gene and protein expression in the heart	[110]
	RL-14 human cardiac-derived cells	10 μM 12 h	Induction of gene and protein expression and catalytic activity	[74]
	RL-14 human cardiac-derived cells	10 μM 24 h	Induction gene and protein expression and catalytic activity	[176]
	H9c2 rat cardiac-derived cells	1–10 μM 2 h	Concentration-dependent induction of CYP1B1 gene expression	[111]
Daunorubicin	Sprague–Dawley male rats	5 mg/kg single dose 1 day post	No change in gene or protein expression in the heart	[169]
Dasatinib	H9c2 rat cardiac-derived cells	0–160 μM for 24 h	Induction of gene expression	[177]
Docetaxel	MDA 453 BT-20 MCF-7 (breast carcinoma)	8 ng/ml 4 h	Induction of gene expression in MDA-453 and BT-20 cells, No change in MCF-7	[178]
Sunitinib	Wistar albino male rats	25, 50, and 100 mg/kg daily for 4 weeks 1 day post	Dose-dependent induction of gene and protein expression in the liver and kidney	[179]
Radiation	Human skin	Ultraviolet B 0–4 minimal erythema doses for 0–48 h	Induction of gene and protein expression in skin biopsies	[180]
	Peripheral blood mononuclear cells	Solar radiation Measured in (W/m^2^) 1 m above the ground for 24 h and given as daily duration (minutes) of the radiation effect exceeding 120 W/m^2^	Significant correlation between solar radiation and CYP1B1 mRNA levels	[181]
	Zebrafish embryos	Ultraviolet B 8.9, 17.9, and 26.8 kJ/m^2^ for 2, 4, and 6 h daily for two consecutive days	Induction of gene expression	[182]
	HaCaT human keratinocytes	Ultraviolet B Dose 20 mJ/cm^2^ for 0–24 h	Induction of CYP1B1 gene transcript	[183]
	HaCaT human keratinocytes	Ultraviolet 0–6.6 mJ/cm^2^ and cultured for 6 h before cell harvest	Induction of protein expression and DNA adduct formation	[184]

The association between CYP1B1 polymorphisms and increased cancer risk has been extensively studied [30]. For instance, in 2015, Li and colleagues conducted a meta-analysis to carry a comprehensive and quantitative analysis on the role of CYP1B1 in cancer [127]. This analysis specifically focused on A453G and G119T, which are two critical polymorphisms that have been associated with the replacement of important amino acids that play a crucial role in catalytic activity. This extensive analysis found a significant association between G119T and A453G with prostate, lung, colorectal, endometrial, breast, bladder, and several other cancer risks [127]. Its polymorphisms, Val^432^Leu, Arg^48^Gly, Ala^119^Ser, and Asn^453^Ser specifically, have been linked to increasing estrogen metabolism responsible for genotoxic metabolites that eventually result in hormone-induced cancers [30]. Additionally, a different meta-analysis focused on several other CYP1B1 polymorphisms. The analysis found that Leu^432^Val polymorphism is associated with ovarian, lung, and endometrial cancer risks. It also found that Asn^453^Ser and Arg^48^Gly are associated with endometrial cancer risks, and Ala^119^Ser is associated with breast cancer risk [135]. In contrast, another analysis found that the CYP1B1 polymorphisms Arg^48^Gly, Ala^119^Ser, and Asn^453^Ser are not associated with breast cancer risk [136]. The mechanisms by which CYP1B1 polymorphisms increase cancer risk include enhanced estrogen and progesterone receptor signaling, also known to influence cancer treatment response [30]. When studying the effect of polymorphisms on chemotherapeutic drug treatments, it was found that polymorphisms induce a slower response to anthracycline agents, whereas low polymorphism levels were shown to improve chemotherapy response [128]. Therefore, the presence of homozygous variant genotype (GG) and variant allele (G) of CYP1B1 4326C>G polymorphism of CYP1B1 was associated with lower response rates, shorter progression-free survival, and an overall decrease in patient survival among patients with triple-negative breast cancer [128]. This study shows the ability of CYP1B1 to interfere with cancer treatments. In an era of precision medicine, cancers with high-activity CYP1B1 variants may better respond to the beneficial effects of CYP1B1 inhibitors.

### CYP1B1 inhibitors

The detrimental role of CYP1B1 in the pathogenesis of cancer and cardiovascular diseases, among other pathologies, has stimulated active research programs to identify and synthesize potent and selective CYP1B1 inhibitors. The medicinal chemistry, classification, and relative potency and selectivity of these inhibitors have been discussed in previously published excellent review articles [137–140]. Phytochemicals, which are chemicals derived from natural plants, have gained great popularity in the pharmaceutical and medicinal applications as potential cardioprotective and chemopreventive compounds due to their anti-inflammatory, antioxidant, anti-angiogenic, anti-mutagenic, and anti-proliferative properties [141–145]. Although not highly selective, phytochemicals have been the most common source for CYP1B1 inhibitors. Phytochemical groups that show strong inhibitory activity and relative selectivity toward CYP1B1 include stilbenes, flavonoids, coumarins, anthraquinones, and alkaloids [26,137]. More selective CYP1B1 inhibitors have been developed, including: 2,4,3′,5′-tetramethoxystilbene which is a highly potent and selective competitive inhibitor of CYP1B1 [146].

Phytochemicals are also of great interest as chemopreventive compounds due to their low toxicity, no apparent side effects, their regulatory role in cell signaling and gene expression, and high tolerance demonstrated in both *in vivo* and *in vitro* studies [142,147]. Flavonoids are among the most common phytochemicals, approximately 6000 different types existing today, found in fruits, vegetables, grains, teas, and wine as well as other beverages [142]. Flavonoids have been suggested for chemoprevention, which may be attributed to their ability to inhibit CYP1B1 expression and activity [26,142,148]. Aside from their potential chemopreventive role in cancer, flavonoids and polyphenolic compounds have also been shown to prevent various other diseases such as obesity, hypertension, and atherosclerosis, possibly via CYP1B1 inhibition [143,144,149]. For instance, a previous study focused on coronary heart disease found that flavonoids provide protective effects such as anti-inflammatory, antithrombotic, anti-ischemic, antioxidant, and vasorelaxant [150]. Moreover, flavonoids have been shown to decrease the risk of coronary heart disease through an improvement of coronary vasodilatation, a decrease in blood clotting in platelets, and a prevention of low-density lipoprotein (LDLs) oxidation [150,151].

Recent efforts have been exerted to discover the CYP1B1 inhibitory activity of commonly used drugs. Intriguingly, the anti-fungal drug fluconazole has been shown to inhibit CYP1B1 and protect against angiotensin II-induced cardiac hypertrophy [152]. Similarly, the clinically relevant β-blocker carvedilol has been found to inhibit CYP1B1 through a systematic drug repurposing approach [153]. Metformin, a medication usually given to treat diabetes, has also been shown to inhibit CYP1B1 expression, specifically in breast cancer cells [154]. Nevertheless, the mechanistic role of CYP1B1 inhibition in mediating the pharmacological effects of these agents is still poorly understood. In addition, almost all inhibitors of CYP1B1 have inhibitory activity toward other members of the CYP1 family, particularly CYP1A1 [155]. Although this lack of selectivity toward CYP1B1 may be undesirable from a mechanistic point of view, it may offer a therapeutic advantage since CYP1A1 is also a reasonable target in cardio-oncology as discussed earlier. Indeed, a number of studies have reported a protective effect of CYP1 inhibitors without discerning the protective effects to either CYP1A1 or CYP1B1 [112,113]. That being said, 2,4,3′,5′-tetramethoxystilbene (TMS) exhibited 50-fold selectivity for CYP1B1 over CYP1A1 and 500-fold selectivity for CYP1B1 over CYP1A2 [146]. Therefore, selective pharmacological inhibition of CYP1B1 can be achieved by using TMS in mechanistic studies. Genetic approaches using Cyp1b1 knockout mice may also be employed to mechanistically discern the exact role of Cyp1b1 [156].

## Anthracycline-induced cardiotoxicity

Anthracyclines (e.g. DOX) are a group of chemotherapeutic agents used to treat hematologic malignancies and solid tumors in both pediatric and adult cancer patients. However, the clinical utility of anthracyclines is limited by a significant anthracycline-induced cardiotoxicity which may progress to end-stage heart failure [157,158]. Indeed, the cardiotoxic effects of anthracyclines were reported in cancer patients as early as the 1970s [159,160]. Anthracyclines have both acute and chronic cardiovascular toxic effects. Acute cardiotoxicity occurs in up to 11% of patients during or soon after the administration of anthracyclines and include various arrhythmias, hypotension, and acute heart failure [161,162]. On the other hand, chronic anthracycline-induced cardiotoxicity is dose-dependent and results in irreversible cardiomyopathic changes that affect approximately 2% of anthracycline-treated patients [163]. The precise mechanism of anthracycline-induced cardiotoxicity has not been fully elucidated yet, despite more than 40 years of research. There are different proposed mechanisms including: increased ROS, mitochondrial dysfunction, apoptotic cell death, altered molecular signaling, and perturbed myocardial energy metabolism [161,164–166].

### Effect of anthracyclines on CYP1B1 expression

*In vitro* and *in vivo* studies have demonstrated the induction of CYP1B1 by DOX (Table 2). We first reported that DOX induced CYP1B1 gene expression in H9c2 cardiomyoblasts [111], an effect that was confirmed in RL-15 human cardiomyocytes at the gene, protein, and catalytic activity levels [74]. We were also the first to report that acute DOX administration induced CYP1B1 in the heart, liver, and kidney of male Sprague–Dawley rats [110,167]. Chronic DOX toxicity has also been shown to induce CYP1B1 in the heart, liver, and kidney [74,168]. Importantly, DOX-mediated induction of CYP1B1 was associated with a significant increase in mid-chain HETEs metabolites in the heart of male rats [74]. Intriguingly, we have recently demonstrated a sex-dependent induction of *Cyp1b1* gene expression by acute DOX administration in male C57Bl/6 mice, but not in female mice [19]. This male-specific induction of *Cyp1b1* was associated with a significant sexual dimorphism with male-specific cardiotoxicity [19]. While DOX has been shown to induce CYP1B1 expression *in vivo* and *in vitro*, a study using another anthracycline, daunorubicin, showed no changes in CYP1B1 gene or protein expression in the heart of male Sprague–Dawley rats [169].

**Figure 2:**
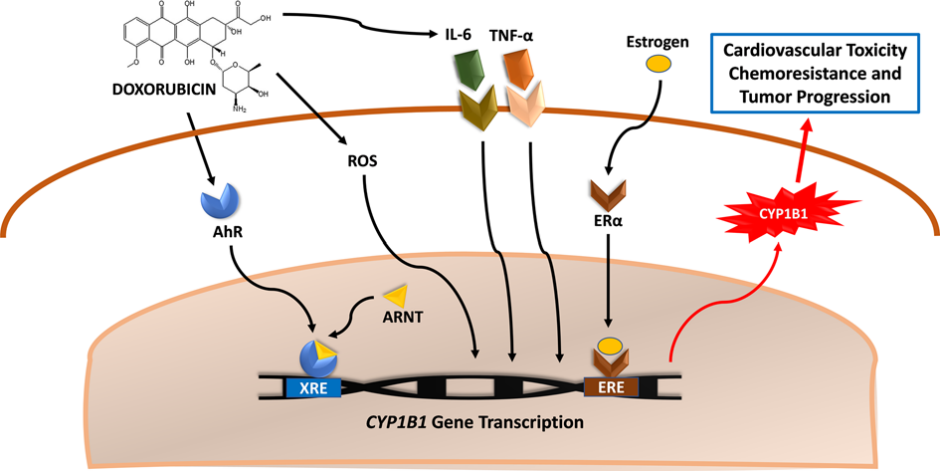
Possible mechanisms of DOX-mediated induction of CYP1B1 DOX may induce CYP1B1 via different mechanisms. First, DOX may directly or indirectly activate the AhR. Upon its nuclear translocation and binding to the AhR Nuclear Translocator (ARNT), the AhR–ARNT heterodimer activates the XRE to induce *CYP1B1* gene transcription. DOX may also induce CYP1B1 by generating ROS and eliciting an inflammatory response via IL-6 and TNF-α. Estrogen can also induce CYP1B1 gene expression via ERα; however, the role of DOX in this pathway is not known. Induction of CYP1B1 leads to both cardiovascular toxicity and increased chemoresistance.

Nevertheless, the aforementioned studies have not precisely defined the mechanism of CYP1B1 induction by DOX. Studying the general mechanisms of CYP1B1 induction, we can speculate that DOX may induce CYP1B1 via AhR activation, ROS generation, and/or inflammatory cytokines production (Figure 2). DOX has been shown to activate the AhR in hearts of C57Bl/6 mice, leading to an induction of Cyp1a1 [170]. Although the effect of DOX-induced AhR activation on Cyp1b1 expression was not reported in that study, it is inferred that DOX induced CYP1b1 since it is an AhR-dependent gene, similar to Cyp1a1. The authors have attributed the DOX-induced AhR activation to binding of DOX to the AhR due to its planar structure that resembles polyaromatic hydrocarbon receptors [170]. Counterintuitively, DOX-induced cardiotoxicity was exacerbated in AhR knock-out mice [170]. The cytoprotective effect of the AhR may be attributed to its intricate interplay with other signaling pathways in the heart, rather than its role in cytochrome P450 regulation. In contrast, DOX-induced apoptosis in H9c2 cardiomyoblasts was ameliorated by ginsenoside Rb1 via inhibition of the AhR pathway [171]. DOX has also been shown to generate a copious amount of ROS, particularly in the heart [172]. These ROS have been shown to induce CYP1B1 as well [173]. At last, DOX has been shown to provoke a strong inflammatory response which may lead to CYP1B1 induction, particularly through IL-6 and TNFα-mediated signaling [167,174]. Intriguingly, DOX-induced inflammation in the heart of C57Bl/6 mice was sexually dimorphic with stronger inflammatory response in hearts of male mice [19]. This was associated with male-specific induction of *Cyp1b1* gene expression, strongly suggesting an important role of inflammation in DOX-mediated up-regulation of CYP1B1 [19].

### Cardioprotective effects of CYP1B1 inhibitors

Protection from anthracycline-induced cardiotoxicity has been provided by several natural compounds with CYP1B1 inhibitory activity both *in vitro* and *in vivo* (Table 3). It is important to mention that these compounds are not selective inhibitors to CYP1B1 and they have multiple other targets that may mediate their cardioprotective effects. Nevertheless, the CYP1B1 selective inhibitor TMS has been shown to protect from chronic DOX-induced cardiotoxicity in male Sprague–Dawley rats *in vivo* and in RL-1 cardiomyocyte-like cells *in vitro* [74]. As summarized in Table 3, the cardioprotective effects of CYP1B1 inhibitors have been shown to be mediated by reduction in oxidative stress and apoptosis [185–188], improving mitochondrial function [189], reversing altered energy metabolism [190], protection from DOX-induced senescence in vascular smooth muscle cells [191], and reducing mid-chain HETEs concentration [74].

**Table 3:** Cardioprotective and chemosensitizing effects of CYP1B1 inhibitors toward anthracyclines

Inhibitor	Inhibition IC_50_ (nM)	Cardioprotective effects	Chemosensitizing effects
Acacetin	7–14 [219–221]	Not reported	Enhances the chemotherapeutic effect of DOX in non-small-cell lung carcinoma cells [194]
Isorhamnetin	17 [219]	Protection from chronic DOX-induced cardiotoxicity *in vivo* in rats and *in vitro* in H9c2 cells [201]	Potentiates DOX-induced toxicity in MCF-7, HepG2, and Hep2 cancer cells [201]
Chrysin	24–270 [219,220]	Protection from acute and chronic DOX-induced cardiotoxicity *in vivo* in rats [222,223]	Enhanced cytotoxicity of DOX in a spheroid culture model of human lung squamous cell carcinoma [224], BEL-7402/ADM [225], lung cancer A549 cells [192], and human non-small-cell lung cancer cell lines [193]
Apigenin	25 [219]	Attenuated chronic DOX-induced cardiotoxicity in *in vivo* in rats and *in vitro* in rat cardiomyocytes [186–188]	Augmented the cytotoxic effect of DOX against HepG2 cells [203], and DOX-resistant hepatocellular carcinoma cell line BEL-7402/ADM [204,218,226] Reverse chemo-resistance to DOX in DOX–resistant breast cancer cells (MCF–7/ADR) [198]
Kaempferol	47 [219]	Protected from chronic DOX-induced cardiotoxicity *in vivo* in rats and *in vitro* in H9c2 cells [227]	Potentiated the cytotoxic effect of DOX in glioblastoma cells [205]
Quercetin	77 [219]	Protected rat and human cardiomyocytes and H9c2 cells from DOX-induced toxicity *in vitro* [176,188,189,199,228]. Protected from chronic DOX in rats [190,229] and mice [185] *in vivo*. Augmented the cardioprotective effect of losartan against chronic DOX cardiotoxicity [230]	Enhanced DOX anti-cancer effects in xenografts of leukemia P388 cells [185], liver cancer cells [231], 4T1 breast cancer cells [232,233] Reversed chemoresistance to DOX in hepatocellular carcinoma cells [202], breast cancer cells [200,212], prostate cancer cells [206], multidrug-resistant leukemia K562 cells [210] Enhanced chemotherapeutic effect of DOX against human breast cancer cells [195–197,199,234], human colorectal HT29 cancer cell line [208], neuroblastoma cells [235]
Luteolin	79 [219]	Protected against DOX-induced cardiomyocyte toxicity *in vitro* [188] Attenuated acute DOX-induced myocardial lipid peroxidation *in vivo* [236]	Luteolin (10 μM) attenuated the cytotoxic effects of DOX in breast cancer cells MCF-7 cells [237] Luteolin (5 μM) sensitized oxaliplatin-resistant colorectal cancer cell lines HCT116 and SW620 [207] and human lung carcinoma A549 cells [238] to DOX
Genistein	IC_50_ = 2100 nm [239] Ki = 1900 nm [240] Induced CYP1B1 gene expression [241,242]	Protected from chronic DOX-induced cardiotoxicity *in vivo* [243,244] Protected from DOX-induced senescence in vascular smooth muscle cells [191]	Potentiated the cytotoxic effect of DOX in MCF-7, MCF-7/ADR cells, MDA-MB-231 (breast), PC-3 (prostate), H460 (lung), and BxPC-3 (pancreas) cancer cells [213,245–247] Attenuated DOX-induced cytotoxicity in MCF-7 breast cancer cells in one study [248] Sensitized diffuse large cell lymphoma to CHOP (cyclophosphamide, DOX, vincristine, prednisone) chemotherapy in SCID tumor-bearing mice *in vivo* [249]
Resveratrol, reviewed in [250]	1400–40000 [251,252]	Protection from DOX-induced cardiomyocyte toxicity in H9c2 cells [253–258], rat primary cardiomyocytes [259–261], and human cardiac progenitor cells [262] *in vitro*. Protection from acute DOX-induced cardiotoxicity [255,263–267] and chronic DOX-induced cardiotoxicity *in vivo* [258,259,262,268–276]	Potentiated DOX-induced cytotoxicity in U373MG glioblastoma, MCF-7 breast cancer cells, LNCaP prostate carcinoma, Reh B-cell leukemia cells, Human ovarian cancer cells OVCAR-3 and uterine (Ishikawa) cells, Human hepatocellular carcinoma cell line (HepG2), Cervical cancer cell line (HeLa), MDA-MB-231 cells, HT-29 human colon carcinoma cells, Hela and Caski cells, HCT 116 and HT-29, Lymphoblastic leukemia cell line (MOLT-4), Human multiple myeloma cell line (U266B1), Burkitt’s lymphoma cell line (Raji cell), canine hemangiosarcoma cells [259,277–286] Reversed chemoresistance in DOX-resistant MCF-7 [214–217], DOX-resistant gastric cancer cells (SGC7901/DOX) [209] Augmented the chemotherapeutic effect of DOX tumor-bearing mice *in vivo* [209,215,217,285,287].
Berberine	Ki = 44, IC_50_ = 90–190 [288,289] Induced CYP1B1 gene expression [290]	Protection from acute DOX-induced cardiotoxicity [291–294] and chronic DOX-induced cardiotoxicity [295] *in vivo* Protection from DOX-induced toxicity in H9c2 cells [291,296] and primary rat cardiomyocytes [294] *in vitro*	Enhanced sensitivity to DOX in Jurkat, HeLa, and lung cancer cells *in vitro* and in leukemia mouse model *in vivo* [211,297,298]. Reversed DOX resistance in resistant human breast cancer MCF-7/MDR cell *in vitro* and *in vivo* [299]. Berberine in combination with DOX suppresses growth of murine melanoma B16F10 cells in culture and xenograft [300]
2,4,3′,5′-tetramethoxy-stilbene	IC_50_ = 6 [146]	Protection from chronic DOX-induced cardiotoxicity in rats *in vivo* and in RL-14 cardiomyocyte-like cells *in vitro* via decreasing the formation of cardiac mid-chain HETEs [74]	Not reported

### Chemosensitizing effects of CYP1B1 inhibitors

CYP1B1 inhibitors have also been shown to enhance the chemotherapeutic effects of DOX in several cancer cell lines including lung cancer [192–194], breast cancer [195–201], liver cancer [201–204], glioblastoma [205], prostate cancer [206], colorectal cancer [207,208], gastric cancer [209], and leukemia [210,211]. Importantly, several inhibitors have also been shown to overcome DOX resistance in DOX-resistant cancer cell lines [209,210,212–217]. Although all these compounds (Table 3) are known inhibitors of CYP1B1, the role of CYP1B1 in mediating the chemosensitizing effects of these compounds have not been determined in the summarized studies. The chemosensitizing effects of these compounds have been attributed to other mechanisms including: AMPK activation to promote cell apoptosis [192], regulating miR-520b/ATG7 axis [204], miR-101/Nrf2 pathway [218], FZD7/β-catenin pathway [202], down-regulating P-glycoprotein (P-gp) expression [200], and the PTEN/Akt pathway [197]. Inhibition of CYP1B1 may interplay with these pathways leading to the chemo-sensitizing effects.

## Radiation therapy

Radiation therapy is a highly effective treatment for many types of cancers including lymphoma, breast, lung, neck, and head cancers [5,301]. However, radiation-based cancer treatments can also result in serious cardiotoxic side effects, including pericardial fibrosis, pericardial effusion, and diffuse myocardial fibrosis, all of which can lead to heart failure [302]. Restrictive cardiomyopathy, valvular abnormalities, coronary disease, peripheral vascular disease, and arrhythmias can also occur following radiation therapy [303]. It has been clearly shown that the risk of heart failure following radiation therapy for various cancers is dose-dependent [304]. In fact, several studies showed a correlation between an increase in radiation dose with an increase in incidence of major coronary events, associating these with cardiac mortality [305,306]. The cardiotoxic effects of radiation therapy may be exhibited 5–30 years following treatment [5,302]. These late-onset cardiac effects are especially observed in patients that have been treated for breast carcinoma, Hodgkin’s lymphoma, lung carcinoma, and other thoracic malignancies, likely due to the incidental irradiation of the heart [304].

### Effect of radiation on CYP1B1 expression

Although there is limited evidence regarding the effect of radiation therapy on CYP1B1 expression, exposure to ultraviolet (UV) and solar radiation have been shown to induce CYP1B1 (Table 2). In a longitudinal study, levels of CYP1B1 mRNA isolated from human peripheral blood mononuclear cells were compared with yearly solar radiation records, and a significant correlation was found [181]. However, this study had several limitations including small sample size and lack of individual radiation exposure levels. Exposure to UV and UV-B radiation induces CYP1B1 mRNA in human keratinocytes, HaCaT cells, zebrafish, and human skin biopsies (Table 2).

### Protective effects of CYP1B1 inhibitors against radiation-induced toxicity

There is a paucity of research showing the protective effects of CYP1B1 inhibitors against radiation therapy-induced cardiovascular toxicity. However, inhibition of CYP1B1 has been shown to protect from other radiation-induced toxicities in non-cardiovascular tissues and organs, including: protection from macromolecular damage, hemorrhage, and fibrosis in HaCaT cells and ovarian tissues by isorhamnetin [219,307], protection against follicular loss and destruction of ovarian histoarchitecture in ovarian tissues by chrysin [308], protection against nuclear DNA damage in HaCaT cells by apigenin [309]. Similarly, resveratrol has been shown to protect experimental animals from radiation-induced erectile dysfunction, immune-suppression, intestinal injury, hepatotoxicity, and ovarian toxicity [310–312]. Berberine has also been shown to reduce the incidence and severity of acute intestinal symptoms in patients receiving pelvic radiation [313]. *In vivo* studies in mice showed the protective effects of berberine against radiation-induced intestinal injury by decreasing inflammation markers, lipid peroxidation, and mucosal injury in the intestinal tissue [314,315]. Moreover, berberine decreased markers of endothelial dysfunction and reduced the incidence of lung injury induced by radiation therapy in patients with non-small cell lung cancer [316]. Several phytochemicals, such as isorhamnetin, chrysin, apigenin, luteolin, berberine, and luteolin have all been shown to protect human keratinocytes from radiation-induced damage through reduction in ROS production [309,317–319].

### Radiosensitizing effects of CYP1B1 inhibitors

While there is limited evidence of the cardiovascular protective effects of CYP1B1 inhibitor against radiation-induced cardiovascular toxicity, there is a plethora of preclinical studies showing the radiosensitizing effects of phytochemicals with CYP1B1 inhibitory activity. While these phytochemicals have been shown to target multiple pathways, they exhibit strong inhibitory activity toward CYP1B1, with IC_50_ values in the nanomolar to micromolar range (Table 3). When combined with radiation therapy, resveratrol has been shown to augment the anti-cancer effects of radiation in both *in vitro* and *in vivo* studies [320–324], as reviewed in [250]. For instance, resveratrol has proven in the past to offer radiosensitizing effects in nasopharyngeal cancer cells via inhibition of E2F transcription factor, colony-forming activities, and the induction of G_1_ phase cell cycle arrest [325]. Apigenin has been shown to enhance the apoptotic effects of radiation in SQ-5 human lung carcinoma cells by increasing the protein expression of WAF1/p21 while decreasing protein levels of Bcl-2 [326]. Moreover, apigenin alongside genistein and quercetin enhanced radiation-induced cell death by decreasing DNA damage renewal and cell repopulation, demonstrating higher antitumor activities [327]. Additional *in vivo* studies also demonstrated the radiosensitizing effects of apigenin in Ehrlich carcinoma-bearing mice exposed to whole body γ irradiation via the down-regulation of angiogenic regulators such as vascular endothelial growth factor-C (VEGF-C), down-regulation of matrix metalloproteinase-2 (MMP2), and the enhancement of apoptosis [328]. The radiosensitizing effects of quercetin have also been demonstrated in DLD-1 human colorectal cancer xenograft model *in vivo* and in HeLa and MCF-7 cells *in vitro* [329,330]. Similarly, berberine has been shown to radiosensitize human esophageal cancer cells (ESCC) at doses lower that 15 µM [331] through down-regulation of RAD51, an important factor whose down-regulation is crucial, as it is found in excessive amounts in ESCCs [331]. Berberine has also been demonstrated to radiosensitize human colon cancer cells via induction of AMPK activation, a protein responsible for the regulation of tumor progression and metastasis and also via decreasing migration of SW480 and HCT 116 cells [332]. Berberine has been shown to radiosensitize human liver cancer cell lines SMMC-7221 exposed to radiation, in which decreased cell viability and tumor growth inhibition were observed in nude mice xenograft [315]. At last, nasopharyngeal carcinoma cells CNE-2, hepatocellular HCC cells, and non-small cell lung cancer cell LLC and A549 are some other cell lines in which berberine has shown to enhance radiosensitivity effects through reduction in proliferation and viability, induction of apoptosis and cell cycle arrest in G_0_ and G_1_ phases, decrease in protein expressions of Sp1, and inhibition of growth factor transforming growth factor-beta (TGF-B) and vimentin proteins [333–335].

## Other cardiotoxic cancer treatments

### Cisplatin

Cisplatin is a chemotherapeutic alkylating agent mostly used to treat ovarian, testicular, lung, and bladder cancers [336,337]. The two most common adverse effects of cisplatin are nephrotoxicity and ototoxicity; nevertheless, cisplatin treatment may also result in severe cardiotoxicic effects including electrocardiographic changes in the heart, acute coronary ischemia [338], arrythmias, myocarditis, cardiomyopathy, and congestive heart failure [339]. The protective effects of CYP1B1 inhibitors against cisplatin-induced cardiovascular damage are not well-studied. However, protection from other cisplatin-induced toxicities have been reported. For instance, chrysin offers protection against cisplatin-induced hepatotoxicity and colon toxicity [340]. Luteolin, kaempferol, chrysin, and quercetin also prevent ototoxicity and nephrotoxicity damage induced by cisplatin [340–345]. Resveratrol offered protection against cisplatin-induced epididymal toxicity, testicular toxicity, and toxicity in ovarian and cavity cancer cells [346,347]. At last, berberine has been shown to reverse the nephrotoxic and hepatotoxic effects caused by cisplatin [348].

Not only do these phytochemicals offer protection against cisplatin-induced toxicity, but they also augment the chemotherapeutic effects of cisplatin by enhancing cell death via induction of apoptosis and/or necroptosis [340,348–356]. For instance, apigenin, specifically targets mTOR/PI3K/Akt signaling pathways to promote the cytotoxic effect of cisplatin by increasing the inhibitory effects on cell migration [354]. Berberine also has the potential to down-regulate the overexpressed genes in squamous cell carcinoma [357]. Isorhamnetin has been shown to trigger microtubule distortion and depolymerization and inhibit cancer cell migration [349]. Since these phytochemicals have multiple molecular targets, the evidence of CYP1B1 involvement in these effects is anecdotal. However, other studies have offered more direct evidence of CYP1B1 in chemoresistance to cisplatin therapy. Immunohistochemistry showed CYP1B1 to be up-regulated in non-small cell lung cancer tissues of cisplatin-resistant patients, and CYP1B1 silencing significantly decreased CXCR4 expression levels and overall cisplatin resistance [358]. To further support these findings, a study using human HEK293 kidney cells found that two potent CYP1B1 inhibitors, 7k (DMU2105) and 6j (DMU2139) with IC_50_ values of 10 and 9 nM, were shown to overcome cisplatin resistance in CYP1B1-overexpressing lines [359]. A third study using *Glycyrrhiza glabra* extract and quercetin, both showing CYP1B1 inhibitory activity, reversed cisplatin resistance in triple-negative MDA-MB-468 breast cancer cells via inhibition of cytochrome P450 1B1 enzyme (CYP1B1) [358,360].

### Cyclophosphamide

Cyclophosphamide is another alkylating chemotherapeutic agent used to treat a variety of cancers. At high doses, cyclophosphamide has been reported to cause cardiotoxic effects [361] that are usually manifested in the forms of myocyte damage, edema, and hemorrhagic necrotic perimyocarditis [362,363]. In contrast with other chemotherapeutic agents, cyclophosphamide has been shown to inhibit CYP1B1 gene expression in HL-6 human acute promyelocytic leukemia cell line [175]. Studies using the flavonoids chrysin and resveratrol proved their ability to exhibit ameliorative effects against brain, heart, liver, testis, kidney, and hepatorenal toxicities induced by cyclophospamide [364,365]. Similarly, apigenin exhibits great inhibitory effects on genotoxicity of antitumor agents. Moreover, cardiotoxicity, hepatotoxicity, gentotoxicity, urotoxicity, and ovarian toxicity effects all seemed to be reduced by quercetin, berberine, and genestein treatment, generally through antioxidant and anti-inflammatory activities [366–368].

### Carfilzomib

Carfilzomib is a chemotherapeutic agent used primarily for the treatment of multiple myeloma [369]. Carfilzomib has been shown to cause cardiotoxic effects such as congestive heart failure, hypertension, coronary artery disease, ischemic heart disease, arrhythmia, and cardiorespiratory arrest [370–372]. Currently, there is no published research that shows the effect of carfilzomib on CYP1B1 expression. There is also a paucity of research describing the cardioprotective effects by CYP1B1 inhibitors. However, there is one study that reported the anti-cancer effect of carfilzomib when used in combination with resveratrol. Resveratrol enhanced the effects of carfilzomib in multiple myeloma cell lines showing higher anti-proliferative and apoptotic effects in a dose-dependent manner [373].

### Dasatinib

Dasatinib, an orally administered chemotherapeutic drug, is an inhibitor of many tyrosine kinases, and is an effective agent for treating chronic myeloid leukaemia [374]. Dasatinib has been shown to induce several adverse effects including pulmonary and cardiovascular toxicities. Dasatinib-induced cardiovascular toxicity may lead to heart failure, pericardial effusion, left ventricular dysfunction, pulmonary artery disease, myocardial ishcemia–reperfusion injury, and pulmonary artery disease [375,376]. Intriguingly, dasatinib has been shown to induce CYP1B1 expression in H9c2 cells, an effect that was associated with an induction of cardiac hypertrophy markers such as B-type natriuretic peptide (BNP) and β-MHC [177]. However, co-treatment with resveratrol did not ameliorate dasatinib-induced expression of these hypertrophic markers [177].

### Sunitinib

Sunitinib is a tyrosine kinase inhibitor commonly used to treat stromal tumors, renal carcinoma, and pancreatic neuroendocrine tumors [377]. Sunitinib-induced cardiotoxic effects have been reported in patients including hypertension, left ventricular systolic dysfunction, and congestive heart failure [378,379]. The mRNA and protein expression levels of CYP1B1 in rat renal and hepatic tissues were induced by sunitinib [179]. Sunitinib has also been shown to activate the AhR/CYP1A1 pathway in rat heart and the cardiac-derived H9c2 cells [380]. Similar to CYP1A1, CYP1B1 is an AhR-regulated gene, so it is expected that CYP1B1 is also induced by sunitinib, although the effect of sunitinib on CYP1B1 was not reported in this particular study. Importantly, resveratrol has been shown to protect from sunitinib-induced cardiac hypertrophy in rats [380]. In contrast with the presumably protective effect of phytochemical inhibitors of CYP1B1, genistein, the most prevalent phytoestrogen in soy, increased sunitinib-induced apoptosis in neonatal rat ventricular myocytes and exacerbated sunitinib-induced lethality in mice [381]. The detrimental effect of phystoestrogens in sunitinib-induced cardiotoxicity can be attributed to the fact that estrogen exacerbates sunitnib-induced cardiotoxicity in female mice [382], in contrast with DOX-induced cardiotoxicity which preferentially affect male mice, as reviewed in [383].

### Immunotherapy

Cancer immunotherapy has emerged as a novel and effective approach to combat incurable cancers by activating the host’s immune system to recognize and destroy the tumor cells [384,385]. Expectedly, activation of the immune system leads to several immune-related adverse effects, including cardiovascular toxicity [386]. Immunotherapy-induced cardiovascular toxicity is mostly inflammatory in nature and includes myocarditis, pericarditis, and vasculitis [387]. Although there are no published reports describing the effect of cancer immunotherapy on CYP1B1 expression, immunotherapy-induced inflammatory reaction is expected to up-regulate CYP1B1. Likewise, there are no published studies reporting the potential protective effects of CYP1B1 inhibitors on immunotherapy-induced cardiovascular toxicity. Nevetheless, natural comounds with CYP1B1 inhibitory activity have demonstrated immunomodulatory functions *in vitro* and *in vivo*, which may contribute to their anti-cancer effects, as recently reviewed [388,389]. Therefore, more research is needed to understand the potential interplay between CYP1B1 inhibitors and cancer immunotherapy in the context of cardio-oncology.

## Conclusions

CYP1B1 has been described as “a unique gene with unique characteristics” because it is implicated in a wide variety of pathological conditions [390]. CYP1B1 plays a central role in the metabolism of several biologically active endogenous compounds (Figure 1). It is also capable of generating carcinogenic metabolites leading to DNA adduct formation, in addition to its role in generating ROS. Therefore, the biological significance of CYP1B1 has been the focus of scientific research of several research groups all over the world. The detrimental role of CYP1B1 in the four phases of carcinogenesis, the initiation, promotion, progression, and metastasis, has been recognized for almost two decades [391,392]. More recently, the contribution of CYP1B1 to the pathogenesis of cardiometabolic diseases has also been increasingly appreciated [8,92,393]. Since we first reported the induction of CYP1B1 by DOX, the most cardiotoxic chemotherapeutic drug [111], a growing body of evidence has strongly suggested the contribution of CYP1B1 to chemotherapy-induced cardiovascular toxicity. All the studied cardiotoxic chemotherapies, with the notable exception of cyclophosphamide, have been shown to induce CYP1B1 in different experimental conditions (Table 2). Induction of CYP1B1 can be detrimental in many ways. First, it can induce or exacerbate therapy-induced cardiovascular complications. Second, it can also lead to significant chemo- and radio-resistance, undermining both the safety and effectiveness of cancer treatment.

**Figure 3:**
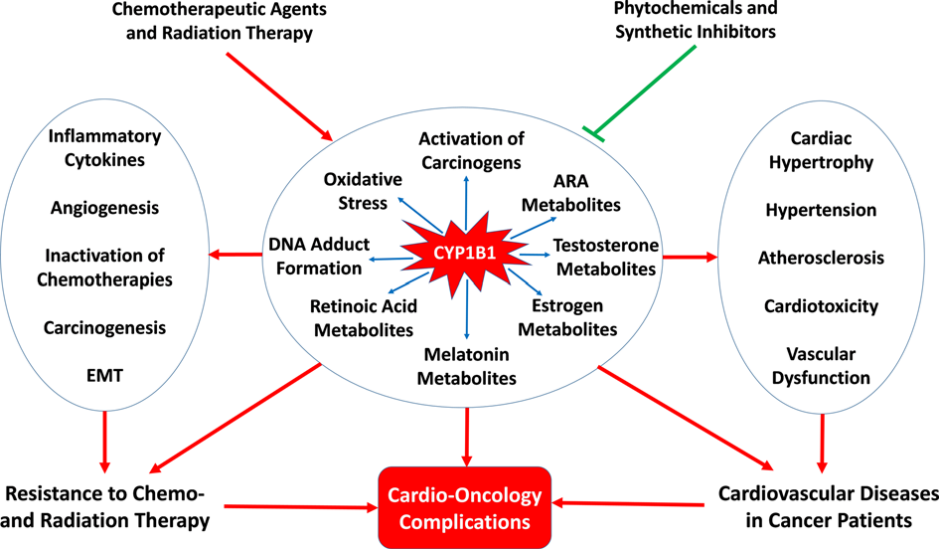
The potential role of CYP1B1 in cardio-oncology Chemo- and radiation therapy induce CYP1B1, leading to perturbation in the metabolism of arachidonic acid (ARA), steroids, melatonin, and retinol, and activation of pro-carcinogens, production of oxidative stress, and DNA adduct formation. Induction of CYP1B1 induces and/or exacerbates therapy-induced cardiovascular toxicity and increases resistance to chemo- and radiation therapy. These detrimental effects can be potentially mitigated by phytochemical and synthetic CYP1B1 inhibitors.

It is intriguing that the same enzyme may have divergent effects on the cardiovascular system and the malignant tumors (Figure 3). Several CYP1B1-mediated signaling pathways may lead to these divergent effects. For instance, while mid-chain and terminal HETEs are detrimental to the cardiovascular system [72,73], they enhance survival, proliferation, and metastasis of cancer cells [394–398]. Likewise, CYP1B1-mediated formation of genotoxic metabolites and DNA adducts lead to atherosclerosis and cardiovascular disease [100,101], and may also contribute to CYP1B1-mediated carcinogenesis [134]. CYP1B1 has also been shown to induce EMT which is involved in cardiac fibrosis [399,400] and in cancer progression [126,401]. CYP1B1-mediated inflammation, which has deleterious effects on the cardiovascular system [19,85,86], can also contribute to carcinogenesis and tumor progression [126,402]. Therefore, inhibitors of CYP1B1 are poised to optimize the benefit and reduce the cardiovascular risk of cancer treatments by interfering with these divergent signaling pathways. Nevertheless, there are no studies that systemically compare these divergent effects within the same model. Indeed, the use of tumor-bearing animal models is strongly needed to discern these divergent signaling pathways underpinning the cardioprotective and the chemo/radio-sensitizing effects of CYP1B1 inhibitors in the same animal model. A plethora of phytochemicals have demonstrated significant CYP1B1 inhibitory activity with varying degrees of potency and selectivity. Although these phytochemicals have shown promising cardioprotective, chemosensitizing, and radiosensitizing properties in preclinical studies, as reviewed in [250,403,404]; the specific role of CYP1B1 inhibition in these effects has been rarely investigated. Since phytochemicals have multiple targets, the identification of a specific molecular mechanism that mediate their effects is very challenging. Therefore, future studies need to discern the role of CYP1B1 by using more selective inhibitors, such as 2,4,3′,5′-tetramethoxystilbene, in addition to CYP1b1 knockout mouse models.

The translation of these promising preclinical findings to the care of cardio-oncology patients is another challenge. A large number of phytochemicals with CYP1B1 inhibitory activity have been tested in clinical trials in healthy individuals, cancer patients, and patients with cardiovascular diseases. The results of these clinical trials are generally mixed and do not provide a strong evidence of a clear clinical benefit. A clinical trial of resveratrol in 20 patients with colorectal cancer has shown a promising anti-cancer effect. Eight doses of 0.5 or 1.0 gram of resveratrol given before surgical resection was well-tolerated and resulted in 5% reduction in tumor proliferation [405]. Likewise, the recurrence rate of neoplasia after colon cancer resection was 7% in patients treated with a flavonoid mixture and 47% in the control [406]. Oral genistein given 14–21 days before urothelial bladder cancer surgery was well-tolerated and reduced bladder cancer tissue phosphorylated-epidermal growth factor receptor (EGFR), which contributes to the proliferation and survival of cancer cells [407]. Although these studies, among others, have shown that these phytochemicals are well-tolerated by cancer patients, a Phase II clinical trial of bortezomib with and without high-dose resveratrol (5 grams daily) in multiple myeloma patients was terminated early due to unexpected renal toxicity in the resveratrol arm [408]. Although this safety concern may be specific to multiple myeloma patients who are at an increased risk for renal failure, these results hindered the advancement of resveratrol and probably other phytochemicals to more clinical trials in cancer patients.

In addition, since several agents which had shown promising cardioprotective effects in preclinical studies failed in subsequent clinical trials (e.g. vitamin E and N-acetyl cysteine [409,410]), the clinical community has become more critical of translating preclinical findings to patient care. Indeed, oncologists are usually very concerned about the possibility that cardioprotective agents may undermine the anti-cancer effects of chemotherapy and/or lead to increased incidence of secondary malignancy. This concern is heightened in case of phytochemicals which have multiple targets and exhibit a high probability of significant drug interactions [411–413]. In addition, there has been a concern that phytochemicals with antioxidant properties may scavenge ROS and negatively impact the outcome of ROS-dependent cancer treatments, as reviewed in [414]. Therefore, elucidating the molecular mechanism of the cardioprotective and chemo/radio-sensitizing properties of phytochemicals is pivotal to the design of specific therapeutic agents that are both safe and effective. As discussed in this review, there is growing evidence that CYP1B1 is an attractive target wherein its inhibition may offer protection against cancer treatment-induced cardiovascular toxicity and prevent chemo/radio-resistance at the same time.

## Open Access

Open access for this article was enabled by the participation of University of Minnesota in an all-inclusive *Read & Publish* pilot with Portland Press and the Biochemical Society.

## Abbreviations

AhR: Aryl hydrocarbon receptor
CYP1B1: cytochrome P450 1B1
DOX: doxorubicin
EET: epoxyeicosatrienoic acid
EMT: epithelial–mesenchymal transition
ERα: estrogen receptor α
ESCC: esophageal cancer cell
HETE: hydroxyeicosatetraenoic acid
IL-6: interleukin-6
PPARα: peroxisome proliferator-activated receptor α
ROS: reactive oxygen species
TCDD: 2,3,7,8-tetrachlorodibenzo-*p*-dioxin
TMS: 2,4,3′,5′-tetramethoxystilbene
UV: ultraviolet
